# Validation and modification of simplified Geriatric Assessment and Elderly Prognostic Index: Effective tools for older patients with diffuse large B‐cell lymphoma

**DOI:** 10.1002/cam4.6856

**Published:** 2023-12-22

**Authors:** Xiaoya Yun, Jiefei Bai, Ru Feng, Jiangtao Li, Ting Wang, Yazi Yang, Jingjing Yin, Long Qian, Shuai Zhang, Qingyun Cao, Xiaoxuan Xue, Hongmei Jing, Hui Liu

**Affiliations:** ^1^ Department of Hematology, Beijing Hospital, National Center of Gerontology Institute of Geriatric Medicine, Chinese Academy of Medical Sciences Beijing P.R. China; ^2^ Graduate School of Peking Union Medical College, Chinese Academy of Medical Sciences Beijing China; ^3^ Department of Hematology, Lymphoma Research Center Peking University Third Hospital, Peking University Beijing China

**Keywords:** diffuse large B‐cell lymphoma, early mortality, geriatric assessment, prognosis

## Abstract

Geriatric assessment can aid in optimizing treatment strategies and supportive interventions for older patients with diffuse large B‐cell lymphoma (DLBCL). Fondazione Italiana Linformi has recently introduced novel geriatric assessment tools, simplified Geriatric Assessment (sGA) and Elderly Prognostic Index (EPI), aimed at tailoring the treatment and predicting the outcomes for older patients with DLBCL. The objectives of this study are the validation and possible modification of the sGA and EPI in China. In the study, both sGA and EPI demonstrated the predictive capabilities for overall survival (OS) and early mortality (both *p* < 0.05) in older individuals with DLBCL. Albumin, serving as an independent predictive biomarker for OS (*p* = 0.006), was utilized to adjust the measurements, resulting in the establishment of sGA‐A and EPI‐A. The sGA‐A effectively addressed the shortcomings of the sGA and EPI in predicting PFS and surpassed them in predicting OS and early mortality. Nevertheless, there is insufficient evidence to support the use of sGA and EPI as treatment guidance tools. In conclusion, the modified sGA‐A model proved to be a successful instrument for geriatric assessment of older patients with DLBCL.

## 1 INTRODUCTION

As the most prevalent form of malignant lymphoma, diffuse large B‐cell lymphoma (DLBCL) predominantly affects older adults with a median diagnostic age of 66 years.^1^, ^2^, ^3^ Given the heterogeneity of age‐associated physiological changes and higher occurrence of comorbidities in older adults, it is imperative to employ suitable geriatric assessment tools to predict prognosis and tailor treatment. For older patients with DLBCL, the potential gains of treatment should be balanced against the risks of treatment‐related morbidity and mortality.^4^ Although R‐CHOP remains the standard first‐line treatment, geriatric assessment could aid in the evaluation of patients' suitability for immunochemotherapy.^2^ Using activities of daily living (ADL), instrumental activities of daily living (IADL), Cumulative Illness Rating Scale for Geriatrics (CIRS‐G), and age, comprehensive geriatric assessment (CGA) has been used to predict survival and customize treatment approaches in older adults with DLBCL.^5^ The CGA distinguishes fit, unfit, and frail patients, and the fit patients may benefit from curative intent treatment. For unfit and frail patients, dose‐reduced therapy is feasible. When some patients cannot tolerate anthracyclines, they can be replaced by etoposide or gemcitabine.^6^, ^7^ However, chemo‐free therapy is also a beneficial option for frail patients.^8^ To further optimize geriatric assessment, researchers devised the simplified geriatric assessment (sGA) and Elderly Prognostic Index (EPI) to predict overall survival (OS), progression‐free survival (PFS), and early mortality.^9^, ^10^, ^11^ These easy‐to‐use tools offer benefits in the identification of distinct risk categories and assist in treatment selection.

No gold‐standard geriatric assessment for patients with DLBCL has been established in China. Nonetheless, there remains uncertainty regarding the suitability of the sGA and EPI for Chinese patients. The study investigated the significance of the sGA and EPI in older Chinese adults with DLBCL and made possible adjustments to optimize each model.

## 2 MATERIALS AND METHODS

### 2.1 Patients

To evaluate the prognostic significance of sGA and EPI, a retrospective study was conducted on elderly patients with DLBCL at Beijing Hospital and Peking University Third Hospital. The inclusion criteria for participants in this research were as follows: (a) the histological analysis rendered a diagnosis of DLBCL based on the 2016 WHO classification^12^; (b) the age of the patients ≥65 years at diagnosis. Patients with HIV infection, primary central nervous system lymphoma, transformed DLBCL, high‐grade B cell lymphoma, or a history of other hematological neoplasms were excluded. According to the criteria, patients who were newly diagnosed between December 2007 and December 2020 were included. The follow‐up period ended on June 30, 2021. The sGA was performed through the application of the following instruments: (1) age; (2) ADL, which reflects the behavioral levels of bathing, dressing, toileting, transfer, continence, and feeding; (3) IADL, an indirect evaluation of ability to use telephone, shopping, food preparation, housekeeping, laundry, mode of transportation, responsibility for own medications, and ability to handle finances; (4) CIRS‐G, which estimates the comorbidities scales in all organs/systems (shown in Table S1). Patients were stratified into fit, unfit, and frail groups according to sGA. The EPI was established with sGA, International Prognostic Index (IPI), and hemoglobin (shown in Table S2).^9^ Patients were classified into low‐, intermediate‐, and high‐risk groups following EPI. The clinical characteristics, treatments, and comorbidities of patients were extracted from their medical records. ADL and IADL were collected by questionnaires at diagnosis, which were completed either by patients themselves or their relatives. Chemotherapy regimens were categorized on the actual delivered anthracycline dose for each patient. Patients who received ≥70% of the theoretical dose were grouped as full‐dose (FD) group (including patients who received CHOP, R‐CHOP ± lenalidomide, and R‐CHOPE ± lenalidomide), and those who received <70% of the theoretical dose were grouped as reduced‐dose (RD) group (including patients who received dose‐reduced R‐CHOP ± lenalidomide, dose‐reduced R‐CHOPE, miniCHOP, and R‐miniCHOP ± lenalidomide). Patients who received therapy that did not include anthracyclines were grouped as palliative‐therapy (PT) group (including patients who received R‐COP±lenalidomide, dose‐reduced COP ± R, R‐miniCOP, R‐C, R2, R + BTK inhibitors±lenalidomide, radiotherapy ± R, and lenalidomide). Besides, patients who received R‐COPE, R‐GEMOX, or R‐GDP were classified as FD group. Therapeutic response was defined based on the Lugano classification.^13^ The overall response rate (ORR) was defined as the obtaining of either complete metabolic response (CMR) or partial response. OS was defined as the time from diagnosis of DLBCL to death from any cause. PFS was defined as the time from diagnosis of DLBCL to disease progression. Grades of toxicity were stratified by Common Terminology Criteria for Adverse Events (CTCAE), version 5.0. The study was determined by the Ethics Committee of Beijing Hospital, National Center of Gerontology, and conducted in accordance with the Declaration of Helsinki.

### 2.2 Data analysis

IBM SPSS Statistics for Windows (version 26.0) and R software (version 4.2.1) were used to analyze the data. Chi‐square tests were used to analyze categorical variables, while one‐way analysis of variance was employed for continuous variables. Kaplan–Meier curves were used to assess survival rates, and receiver operating characteristic (ROC) curves and area under curve (AUC) were used to assess the sensitivity and specificity of the sGA and EPI, respectively, whereas the log‐rank test was used for comparison. Univariate and multivariate Cox regression analyses were used to identify the independent biomarkers for the patients. Logistics regression analyses were used to analyze the correlation between the geriatric assessment tools and early mortality. *p*‐values <0.05 were considered statistically significant.

## 3 RESULTS

### 3.1 Clinical characteristics

A total of 257 patients were included in the study. Among them, 51.8% are men. Their median age was 74 years, with a range of 65–95. Additionally, 67 individuals (26.1%) were 80 years or older. In terms of Ann Arbor stage, 176 (68.5%) were at stage III–IV. Based on the sGA scale, 45.1% of patients were classified into the fit group; 35.4% into were unfit group; and 19.5% into were frail group. Scores on the sGA were associated with age (*p* < 0.001), Eastern Cooperative Oncology Group Performance Status (ECOG‐PS; *p* < 0.001), IPI (*p* = 0.002), B symptoms (*p* = 0.002), hemoglobin (*p* < 0.001), and albumin (*p* < 0.001). Due to inadequate data in the medical records of 10 patients, the remaining patients were categorized as low‐ (15.0%), intermediate‐ (40.1%), and high‐risk (44.9%) groups according to the EPI scale. Age (*p* < 0.001), Ann Arbor stage (*p* < 0.001), extranodal sites (*p* < 0.001), ECOG‐PS (*p* < 0.001), lactic dehydrogenase (*p* < 0.001), IPI (*p* < 0.001), B symptoms (*p* < 0.001), hemoglobin (*p* < 0.001), and albumin (*p* < 0.001) exhibited significant correlations with EPI scores.

### 3.2 Therapeutic response

Except for two patients who did not receive therapy for economic reasons, 111 (43.2%) patients received FD therapy, and 85 patients (33.1%) received RD therapy, while 59 patients (23.0%) received PT therapy. The detailed information regarding the therapy distributions is shown in Table 1. Of these, therapeutic responses for 25 patients were absent. Additionally, the analyses did not include five patients who did not receive rituximab. As shown in Table 2, the ORR of older patients with DLBCL was 79.4%. The CMR rate was 61.8%. Chi‐squared analyses revealed that the sGA (*χ* ^2^ = 12.419, *p* = 0.043) and EPI (*χ* ^2^ = 12.472, *p* = 0.042) were related to the therapeutic response of older patients with DLBCL.

**TABLE 1:** Therapies for older patients with diffuse large B‐cell lymphoma (DLBCL).

Therapy	*N*
*Full‐dose therapy*	
R‐CHOP	80
R‐CHOPE	21
CHOP	1
R2‐CHOP	3
R2‐CHOPE	1
R‐COPE	2
R‐GEMOX	1
R‐GDP	2
*Reduced‐dose therapy*	
Reduced‐dose R‐CHOP	44
Reduced‐dose R‐CHOPE	27
Reduced‐dose R2‐CHOP	2
Reduced‐dose R‐COPE	3
Reduced‐dose COPE	1
R‐miniCHOP	6
R2‐miniCHOP	1
miniCHOP	1
*Palliative therapy*	
R‐COP	33
R2‐COP	2
Reduced‐dose R‐COP	3
Reduced‐dose COP	1
R‐miniCOP	3
R‐C	2
R2	5
R2 + BTK inhibitors	4
R + BTK inhibitors	2
R + BTK inhibitors+BCL‐2 inhibitor	2
Radiotherapy	1
Radiotherapy+R	1

**TABLE 2:** Therapeutic responses of older patients with diffuse large B‐cell lymphoma.

Therapeutic responses	sGA				EPI			
	FIT, *N* = 109(%)	UNFIT, *N* = 79(%)	FRAIL, *N* = 40(%)	Total N = 228(%)	Low, *N* = 34(%)	Intermediate, *N* = 91(%)	High, *N* = 93(%)	Total, *N* = 218(%)
OR	93 (85.3)	58 (73.4)	30 (75.0)	181 (79.4)	30 (88.2)	76 (83.5)	64 (68.8)	172 (78.9)
CMR	76 (69.7)	47 (59.5)	18 (45.0)	141 (61.8)	28 (82.4)	60 (65.9)	47 (50.5)	135 (61.9)
PR	17 (15.6)	11 (13.9)	12 (30.0)	40 (17.5)	2 (5.9)	16 (17.6)	19 (20.4)	37 (17.0)
SD	2 (1.8)	3 (3.8)	3 (7.5)	8 (3.5)	0 (0)	3 (3.3)	5 (5.4)	8 (3.7)
PD	14 (12.8)	18 (23.5)	7 (17.5)	39 (17.1)	4 (11.8)	12 (13.2)	22 (23.7)	38 (17.4)

Abbreviations: CMR, complete metabolic response; EPI, Elderly Prognostic Index; OR, overall response; PD, progressive disease; PR, partial response; SD, stable disease; sGA, simplified geriatric assessment.

Based on the sGA category, FD therapy was prescribed to 71.3%, 23.9%, and 6.4% of the fit, unfit, and frail groups, respectively. The ORRs for the fit, unfit, and frail groups were 85.3%, 73.4%, and 75.0%, respectively. The CMR rates for the fit, unfit, and frail groups were 69.7%, 59.5%, and 45.0%, respectively. Therefore, there was a significant difference in the CMR rate among the three groups (*χ* ^2^ = 7.863, *p* = 0.020). As for EPI, FD therapy was prescribed to 89.2%, 52.0%, and 20.8% of the low‐, intermediate‐, and high‐risk groups, respectively. The ORRs for the low‐, intermediate‐, and high‐risk groups were 88.9%, 83.5%, and 68.8%, respectively. The CMR rates of the low‐, intermediate‐, and high‐risk groups were 82.4%, 65.9%, and 50.5%, respectively. There were significant differences in OR (*χ* ^2^ = 6.459, *p* = 0.040) and CMR (*χ* ^2^ = 11.753, *p* = 0.003) among the three groups.

### 3.3 Overall survival

**FIGURE 1:**
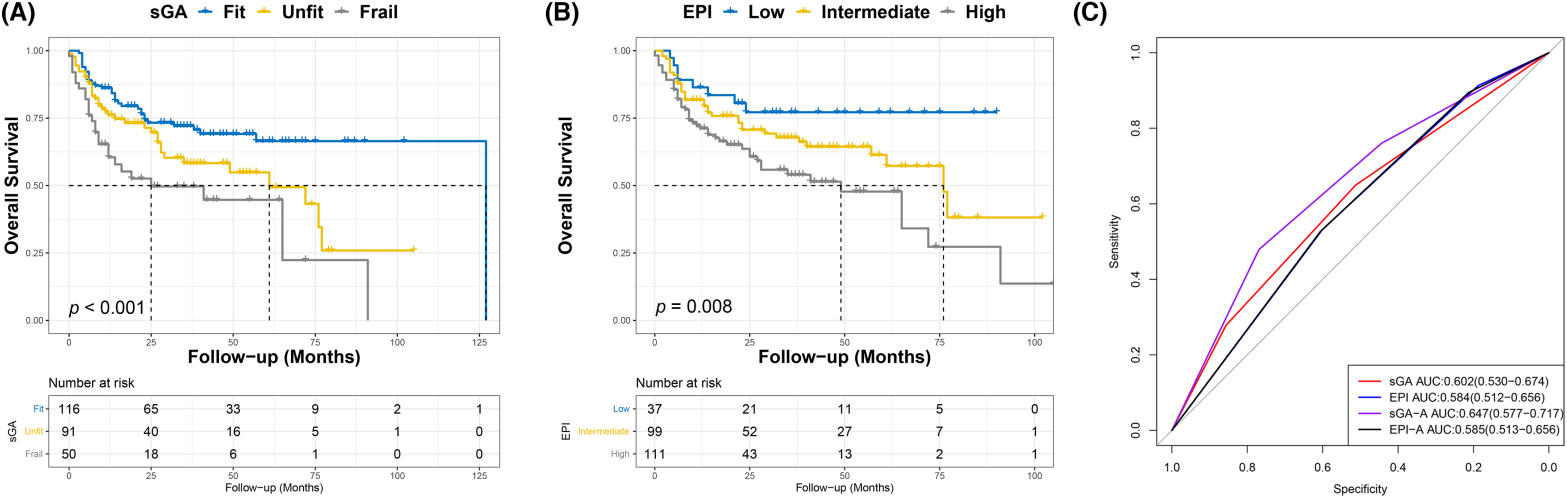
Kaplan–Meier and ROC curves for the sGA and EPI in predicting the OS of older patients with DLBCL. (A) The sGA and (B) EPI were associated with OS in older patients with DLBCL. (C) ROC curves for predicting OS in older patients with DLBCL.

With a median follow‐up time of 23 months (range: 1–127 months), the relationship between sGA (*p* < 0.001) and EPI (*p* = 0.008) was observed in older patients with DLBCL (Figure 1A,B). Applying the sGA to the total cohort, the 5‐year OS rate for the fit, unfit, and frail group was 71.6%, 64.8%, and 52.0%, respectively. According to sGA results, OS rates were significantly different between the fit and frail groups, with a hazard ratio (HR) of 2.60 for frail versus fit (95% CI: 1.46–4.62; *p* < 0.001). In terms of the EPI, the 5‐year OS rate of the low‐, intermediate‐, and high‐risk groups was 78.4%, 67.7%, and 59.5%, respectively. The HR between the low‐ and high‐risk groups was 2.76 (95% CI: 1.54–4.96; *p* = 0.018).

ROC curves suggested that both sGA (*p* = 0.006) and EPI (*p* = 0.027) were statistically significant for OS prediction in older adults with DLBCL (Figure 1C). The AUC for the sGA was 0.602 (95% CI: 0.530–0.674; *p* = 0.006), while that for the EPI was 0.584 (95% CI: 0.512–0.656; *p* = 0.027). However, there was no significant difference between the sGA and EPI in OS prediction (*p* = 0.453).

### 3.4 Progression‐free survival

**FIGURE 2:**
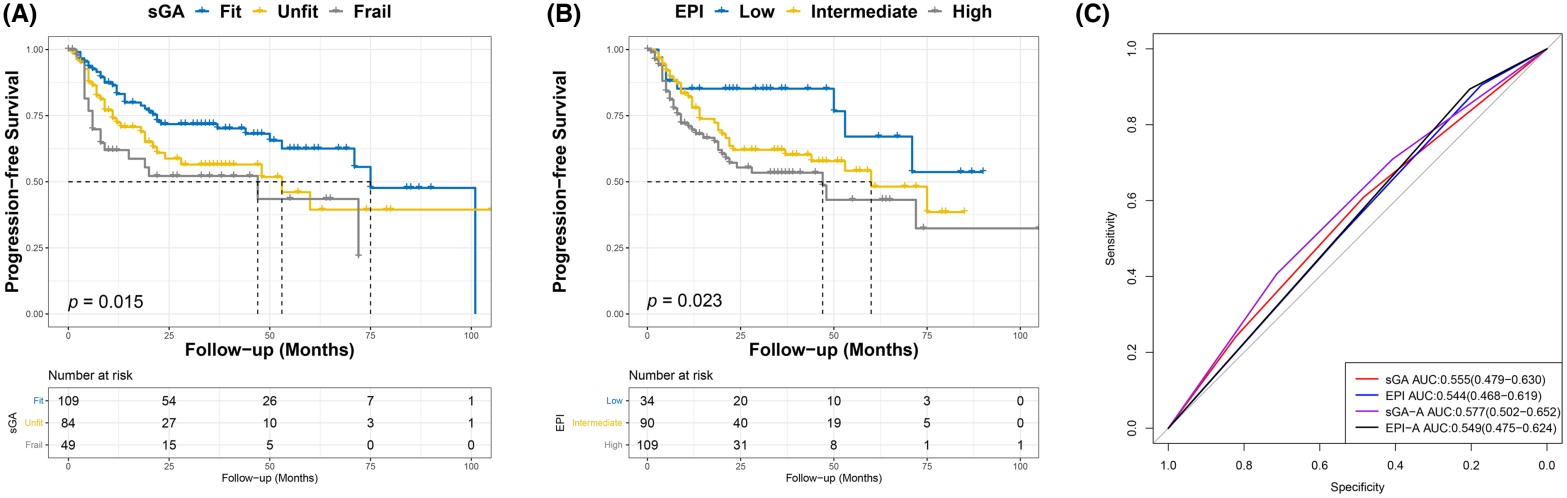
Kaplan–Meier and ROC curves for the sGA and EPI in predicting the PFS of older patients with DLBCL. (A) The sGA and (B) EPI were associated with PFS in older patients with DLBCL. (C) ROC curves for predicting PFS in older patients with DLBCL.

The median PFS time was 71 months (range: 1–105 months). Kaplan–Meier curves showed that sGA was related to PFS in older adults with DLBCL (*p* = 0.015; Figure 2A). Based on the classification of sGA, the 5‐year PFS rate of fit, unfit, and frail group was 71.6%, 61.9%, and 59.2%, respectively. PFS was significantly different between the fit and frail groups, with an HR of 2.14 for frail versus fit (95% CI: 1.16–3.94; *p* = 0.011). EPI was also associated with PFS in older adults with DLBCL (*p* = 0.023; Figure 2B). In the analysis of the EPI, the 5‐year PFS rate of low‐, intermediate‐, and high‐risk was 79.4%, 63.3%, and 62.4%, respectively. The HR between the low‐ and high‐risk groups was 2.66 (95% CI: 1.46–4.82; *p* = 0.029). Regrettably, neither the sGA (*p* = 0.158) nor the EPI (*p* = 0.268) could predict PFS in older patients with DLBCL (Figure 2C).

### 3.5 Early mortality

**FIGURE 3:**
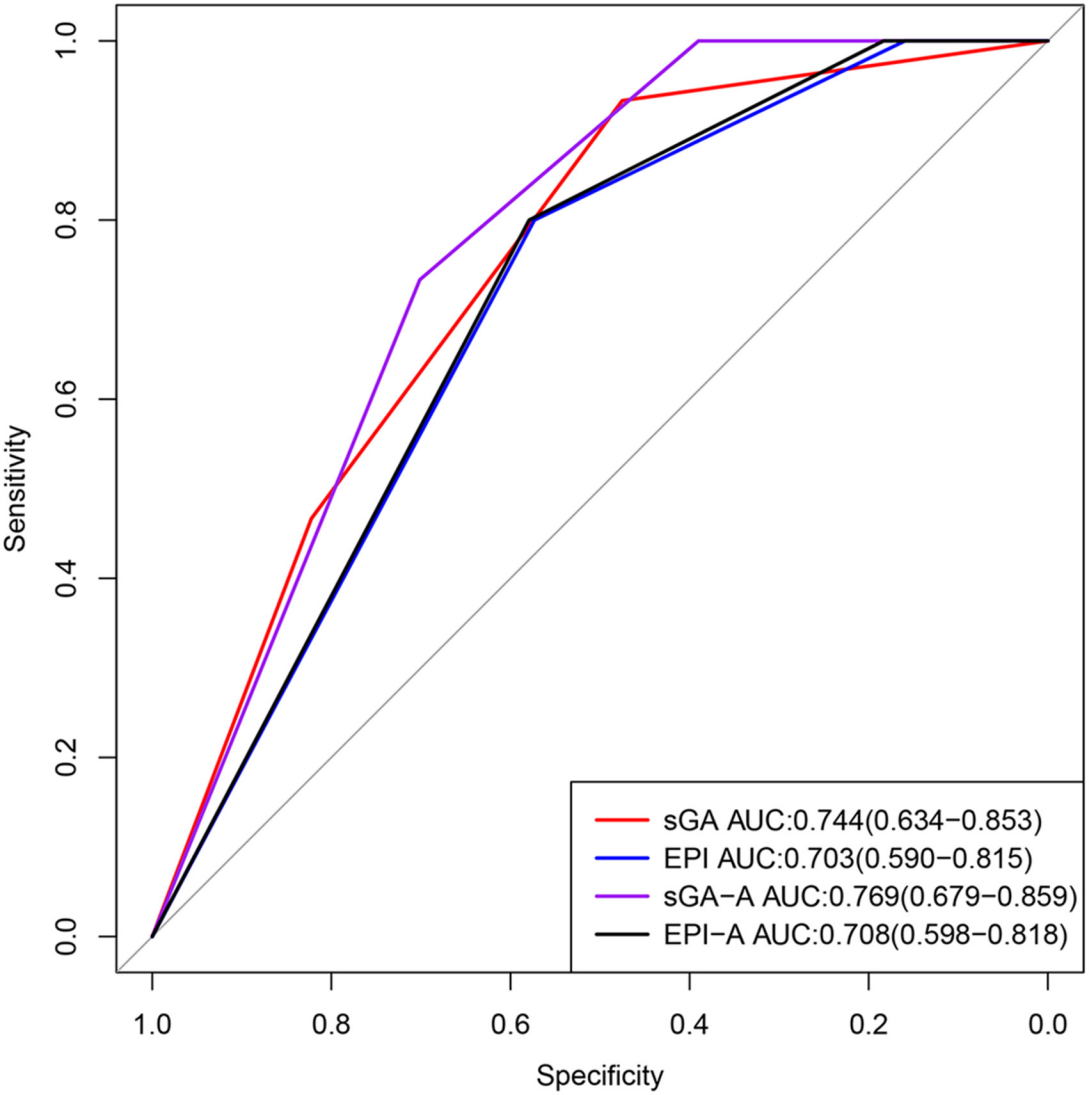
ROC curves for predicting early mortality in older patients with DLBCL.

Among 257 patients, 97 (37.7%) experienced mortality, with 15 (5.8%) of these individuals succumbing to early mortality, defined as death occurring within 3 months of diagnosis.^11^ Among those who experienced early mortality, six (40.0%) deaths were attributed to treatment‐related factors, and three (20.0%) deaths were results of progressive disease. Additionally, one (6.7%) patient died from acute myocardial infarction, and three (20.0%) patients died from Covid‐19. However, the cause of death for two patients was unknown. Logistic regression analyses suggested that sGA (*p* = 0.002; HR: 3.291; 95% CI: 1.570–6.897) and EPI (*p* = 0.011; HR: 4.628; 95% CI: 1.411–15.188) were related to early mortality in older patients with DLBCL. Furthermore, with the AUC of 0.744 (95% CI: 0.634–0.853), sGA could predict the probabilities of early mortality in older adults with DLBCL (*p* = 0.002). EPI also took effect on early mortality prediction in older patients with DLBCL, with an AUC of 0.703 (95% CI: 0.590–0.815; *p* = 0.009) (Figure 3).

### 3.6 Toxicity

The toxicity responses of older patients with DLBCL are presented in Table S3. Hematological toxicity and gastrointestinal adverse reactions were absent in three patients, and cardiovascular adverse reaction was missed in four patients. Approximately 86.2% of patients had hematological toxicity, whereas 46.9% of patients had febrile neutropenia. The incidence of gastrointestinal and cardiovascular adverse reactions was 22.5% and 18.9%, respectively. EPI was related to the incidence of hematological adverse reactions (*p* = 0.004) and neutropenia (*p* < 0.001). However, there was no significant difference in treatment‐related toxicity among the three groups classified using the sGA (*p* > 0.05).

### 3.7 Treatment selection

In the unfit and frail group stratified by sGA, there were 28 (20.7%) patients received FD therapy, and 55 (30.7%) patients received RD therapy. The 5‐year OS rate for FD therapy and RD therapy was 63.8%, and 68.0%, respectively (*p* = 0.617). In the intermediate‐ and high‐risk group based on the EPI category, there were 73 (35.8%) patients received FD therapy, while 79 (38.7%) patients received RD therapy. The 5‐year OS rate for patients who received FD therapy and RD therapy was 63.3%, and 65.4%, respectively (*p* = 0.821). There was no significant difference in toxicities between the group who received FD therapy and RD therapy (*p* > 0.05).

### 3.8 Modification of sGA and EPI

**FIGURE 4:**
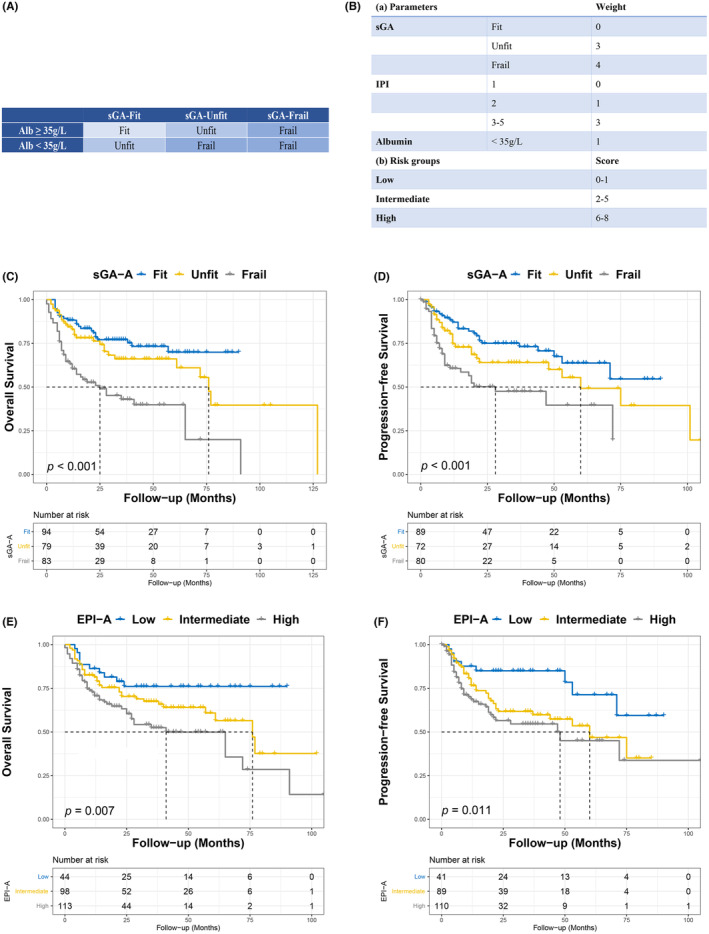
Kaplan–Meier curves for the sGA‐A and EPI‐A in predicting OS and PFS in older patients with DLBCL. (A) The sGA‐A model. (B) The EPI‐A model. The sGA‐A was associated with (C) OS, and (D) PFS in older patients with DLBCL. The EPI‐A was associated with (E) OS, and (F) PFS in older patients with DLBCL.

As mentioned above, the sGA and EPI could predict OS but not PFS in older patients with DLBCL. In addition, the ROC curve showed that the sGA could not predict OS accurately. Thus, taking OS as the endpoint of the main study, Cox regression analyses were performed. Cox analyses revealed that albumin (*p* = 0.006) and ECOG‐PS (*p* = 0.043) were independent prognostic factors of OS in older patients with DLBCL (Table S4). Since assessment tools for physical function, including ADL and IADL, were included in the sGA, ECOG‐PS was not used to modify the sGA and EPI. However, albumin levels were added to develop modified models, termed sGA‐A and EPI‐A, as shown in Figure 4A,B, respectively. The sGA‐A was associated with OS (*p* < 0.001), PFS (*p* < 0.001; Figure 4C,D), and early mortality (*p* = 0.002). The EPI‐A was also associated with OS (*p* = 0.007; Figure 4E), PFS (*p* = 0.011; Figure 4F), and early mortality (*p* = 0.010). With an AUC of 0.647 (95% CI: 0.577–0.717), the sGA‐A was superior to the sGA (*p* = 0.010), EPI (*p* = 0.006), and EPI‐A (*p* = 0.005) in predicting OS (Figure 1C). Besides, the sGA‐A could predict the PFS of older patients with DLBCL (AUC: 0.577, 95% CI: 0.502–0.652, *p* = 0.046). Conversely, the sGA, EPI, and EPI‐A did not exhibit significant predictive capabilities (All *p* > 0.05). Regarding early mortality, the sGA‐A was superior to the EPI (*p* = 0.033) and EPI‐A (*p* = 0.029) but not the sGA (*p* = 0.520) (Figure 3).

In the unfit and frail group based on the sGA‐A category, there were 42 (26.8%) patients received FD therapy, and 62 (36.5%) received RD therapy. The 5‐year OS rate for the group who received FD therapy and RD therapy was 66.2% and 63.2%, respectively (*p* = 0.871). Furthermore, no statistically significant disparities in toxicities were observed between the cohort that underwent FD therapy and the one that received RD therapy.

## 4 DISCUSSION

The current study demonstrated the ability to predict OS and early mortality in older Chinese patients with DLBCL. However, neither sGA nor EPI was found to be predictive of PFS in this patient population. In addition, sGA and EPI were associated with the treatment response in these patients. To optimize geriatric assessment tools in China, we developed modified models, namely sGA‐A and EPI‐A, which incorporated albumin levels. The sGA‐A addressed the limitations of the sGA in predicting PFS and exhibited superior predictive ability for OS and early mortality. Consequently, the modified sGA‐A model may be a feasible geriatric assessment tool for older patients with DLBCL in China.

Previous studies have acknowledged the significance of CGA in DLBCL.^14^, ^15^ When assessing the objective health of older adults across multiple domains, including cognitive impairment, functional or physical impairment, and multimorbidity, geriatric assessment may affect cancer prognosis, treatment choices, and tolerance.^16^, ^17^, ^18^, ^19^, ^20^, ^21^ Notably, the G‐8 screening tool has demonstrated high sensitivity and acceptable specificity, and possess prognostic value in hematological malignancies.^22^, ^23^ The Vulnerable Elders Survey‐13 is a stronger predictor of 1‐year mortality in older patients with DLBCL.^24^, ^25^ Isaksen et al. established a frailty score to aid in treatment‐intensity decisions in older patients with DLBCL.^26^, ^27^ As mentioned above, the oGA can be used to predict OS and PFS in older patients with DLBCL.^5^, ^28^ Additionally, it can be employed to anticipate and manage serious adverse events^29^ and provide guidance for treatment approaches.^30^, ^31^, ^32^, ^33^, ^34^ Summarily, the implementation of geriatric assessment tools aids in the evaluation of the prognosis and tailoring the treatment of elderly patients with DLBCL.

As the sGA and EPI were developed to improve the prognostic assessment of older patients with DLBCL, we validated both assessments in a Chinese cohort. In this study, both the sGA and EPI could predict OS and early mortality but not PFS. In order to improve the predictive ability of OS, we incorporated albumin into the sGA, resulting in a modified geriatric assessment tool known as the sGA‐A. The sGA‐A demonstrated superior performance compared to the sGA and EPI, as it not only predicted PFS but also exhibited better predictive accuracy for OS and early mortality. One of the primary advantages of utilizing sGA‐A as a geriatric assessment tool is its consideration of patients' nutrition status. This aspect is particularly significant as nutrition status exhibits a strong association with diverse metabolic processes and significantly influences the response and prognosis of cancer patients.^35^, ^36^ Decreased levels of albumin may serve as an indicator of the vulnerable condition and inadequate nutritional status among older patients.^37^, ^38^ In addition, albumin was identified as a critical prognostic biomarker for patients with DLBCL.^39^, ^40^, ^41^ Besides, albumin was recommended as a factor to aid in treatment decision‐making.^42^ In the case of low‐risk patients categorized based on their IPI score, albumin levels at the time of diagnosis can identify those who are likely to achieve better treatment outcomes.^43^ As a simplified model, the sGA can be performed by the physicians and takes less than 10 min.^9^ The integration of sGA with albumin, an easily accessible nutritional index, enhances the accuracy of risk stratification through sGA‐A. Consequently, this tool holds potential as a convenient resource for Chinese medical practitioners.

There is currently a limited comprehension regarding the utilization of geriatric assessment‐guided treatment in DLBCL.^4^, ^44^ However, the application of sGA and EPI for determining appropriate therapeutic dosages in older patients with DLBCL has not been explored. It is widely accepted that fit patients would derive benefits from standard‐dose R‐CHOP therapy. Conversely, for unfit/frail patients categorized by sGA or sGA‐A, or intermediate‐/high‐risk patients categorized by EPI, it is evident that those who received anthracycline‐based therapy exhibited longer OS and PFS time compared to those who did not receive anthracyclines. In the cohort, there was an absence of statistically significant disparity in OS between individuals who underwent FD therapy and those who received RD therapy. The finding implies that anthracycline dose‐reduced therapy may be adequate for this particular patient population. Furthermore, no substantiated evidence was found to support the notion that RD therapy mitigates toxicities. The lack of evidence may be attributed to the limited size of the sample and the comparatively poorer physical condition of patients who underwent RD therapy. In summary, dose‐reduced therapy emerges as a more favorable option for patients who are deemed unfit or frail by sGA, as well as those classified as intermediate‐ or high‐risk by EPI.

There were several limitations in our study. Firstly, the sample size was small, which prevented us from dividing the validation cohort to assess the value of sGA‐A. Additionally, the patients included in the study were from Beijing Hospital and Peking University Third Hospital, both of which are renowned for their high‐quality medical care and optimal multidisciplinary teams. Consequently, the prognosis of the patients was significantly better than anticipated due to the selection bias. Therefore, it is imperative to evaluate the impact of sGA‐A in a more extensive cohort, encompassing patients from diverse centers.

## 5 CONCLUSION

In summary, the findings of our study indicate that both the sGA and the EPI exhibit efficacy as prognostic tools for predicting OS and early mortality in older patients with DLBCL. Moreover, the integration of sGA with albumin levels, resulting in the development of sGA‐A, yielded a modified geriatric assessment tool that demonstrated superior performance in predicting OS and early mortality compared to the individual utilization of sGA and EPI. Additionally, sGA‐A also exhibited predictive capabilities for PFS. However, further investigations are warranted to ascertain the optimal utilization of geriatric assessment tools in guiding treatment decisions with enhanced precision.

## AUTHOR CONTRIBUTIONS

**Xiaoya Yun:** Data curation (equal); investigation (lead); methodology (lead); software (equal); visualization (lead); writing – original draft (lead); writing – review and editing (lead). **Jiefei Bai:** Data curation (equal); resources (equal). **Ru Feng:** Data curation (equal); resources (equal). **Jiangtao Li:** Data curation (equal); resources (equal). **Ting Wang:** Data curation (equal); resources (equal). **Yazi Yang:** Data curation (equal); resources (equal). **Jingjing Yin:** Data curation (equal); resources (equal). **Long Qian:** Data curation (equal); resources (equal). **Shuai Zhang:** Data curation (equal). **Qingyun Cao:** Data curation (equal). **Xiaoxuan Xue:** Data curation (equal). **Hongmei Jing:** Data curation (equal); resources (equal). **Hui Liu:** Conceptualization (lead); data curation (equal); funding acquisition (lead); resources (equal); supervision (lead); writing – review and editing (supporting).

## CONFLICT OF INTEREST STATEMENT

None.

## ETHICS STATEMENT

This study was approved by the Ethics Committee of Beijing Hospital, National Center of Gerontology. All participants signed a written informed consent form in accordance with the Declaration of Helsinki.

## Supporting information


Table S1.

Table S2.

Table S3.

Table S4.

## Data Availability

Research data are not shared.
