# Supplementary material for: Validation and modification of simplified Geriatric Assessment and Elderly Prognostic Index: Effective tools for older patients with diffuse large B‐cell lymphoma
Source: Cancer Med. 2023 Dec 22;13(1):e6856. doi: 10.1002/cam4.6856 (PMC10807600; doi:10.1002/cam4.6856)
Supplement: Supplementary file 1 — Table S1. [file CAM4-13-e6856-s003.docx]

**Table S1. The classified criteria of older patients with DLBCL according to sGA.**

| Factor | Fit | Unfit | | Frail |
| --- | --- | --- | --- | --- |
| Age | < 80 | < 80 | ≥ 80 | ≥ 80 |
| ADL | ≥ 5 | < 5 | 6 | 6 |
| IADL | ≥ 6 | < 6 | 8 | < 8 |
| CIRS-G | 0 score = 3-4,  ≤ 8 score = 2 | ≥ 1 score = 3-4,  > 8 score = 2 | 0 score = 3-4,  < 5 score = 2 | ≥ 1 score = 3-4,  ≥ 5 score = 2 |

Abbreviation: sGA, simplified geriatric assessment; ADL, activities of daily living; IADL, instrumental activities of daily living; CIRS-G, Cumulative Illness Rating Scale for Geriatrics.
