# Supplementary material for: Validation and modification of simplified Geriatric Assessment and Elderly Prognostic Index: Effective tools for older patients with diffuse large B‐cell lymphoma
Source: Cancer Med. 2023 Dec 22;13(1):e6856. doi: 10.1002/cam4.6856 (PMC10807600; doi:10.1002/cam4.6856)
Supplement: Supplementary file 2 — Table S2. [file CAM4-13-e6856-s001.docx]

**Table S2. The parameters (A) and the classified criteria (B) of older patients with DLBCL according to EPI.**

| **(A) Parameters** | | **Weight** |
| --- | --- | --- |
| sGA | Fit | 0 |
|  | Unfit | 3 |
|  | Frail | 4 |
| IPI | 1 | 0 |
|  | 2 | 1 |
|  | 3-5 | 3 |
| Hemoglobin | < 12g/dL | 1 |
| **(B) Risk groups** | | **Score** |
| Low | | 0-1 |
| Intermediate | | 2-5 |
| High | | 6-8 |

Abbreviation: EPI, Elderly Prognostic Index; sGA, simplified geriatric assessment; IPI, International Prognostic Index.
