# Supplementary material for: Validation and modification of simplified Geriatric Assessment and Elderly Prognostic Index: Effective tools for older patients with diffuse large B‐cell lymphoma
Source: Cancer Med. 2023 Dec 22;13(1):e6856. doi: 10.1002/cam4.6856 (PMC10807600; doi:10.1002/cam4.6856)
Supplement: Supplementary file 3 — Table S3. [file CAM4-13-e6856-s002.docx]

**Table S3. Toxicities of older patients with diffuse large B-cell lymphoma.**

| **Toxicity** |  | **sGA** | | |  | | **EPI** | | | **Total**  **n/N(%)** |
| --- | --- | --- | --- | --- | --- | --- | --- | --- | --- | --- |
|  | **FIT**  **n/N(%)** | **UNFIT**  **n/N(%)** | **FRAIL**  **n/N(%)** | ***P*-value** |  | **Low**  **n/N(%)** | **Intermediate**  **n/N(%)** | **High**  **n/N(%)** | ***P*-value** |  |
| **Hematological toxicity** | 94/116(81.0) | 81/89(91.0) | 44/49(89.8) | 0.082 |  | 27/37(73.0) | 82/99(82.8) | 101/108(93.5) | 0.004 | 219/254(86.2) |
| **Febrile neutropenia** | 54/116(46.6) | 43/89(48.3) | 22/49(44.9) | 0.925 |  | 13/37(35.1) | 45/99(45.5) | 58/108(53.7) | 0.131 | 119/254(46.9) |
| **Neutropenia** | 80/116(69.0) | 74/89(83.1) | 37/49(75.5) | 0.082 |  | 20/37(54.1) | 71/99(71.7) | 93/108(86.1) | <0.001 | 192/254(75.6) |
| **Anemia** | 93/116(80.2) | 73/89(82.0) | 42/49(85.7) | 0.685 |  | 27/37(73.0) | 78/99(78.8) | 95/108(88.0) | 0.067 | 208/254(81.9) |
| **Thrombocytopenia** | 30/116(25.9) | 21/89(23.6) | 19/49(38.8) | 0.142 |  | 9/37(24.3) | 25/99(25.3) | 32/108(29.6) | 0.734 | 70/254(27.6) |
| **Gastrointestinal toxicity** | 27/116(23.3) | 15/89(16.9) | 14/49(28.6) | 0.258 |  | 8/37(21.6) | 22/99(22.2) | 25/108(23.1) | 0.977 | 56/254(22.0) |
| **Cardiovascular toxicity** | 24/116(20.7) | 13/89(14.6) | 9/48(18.8) | 0.531 |  | 7/37(18.9) | 19/99(19.2) | 20/107(18.7) | 0.996 | 46/253(18.2) |

**Abbreviation:** sGA, simplified geriatric assessment; EPI, Elderly Prognostic Index.
