# Supplementary material for: Validation and modification of simplified Geriatric Assessment and Elderly Prognostic Index: Effective tools for older patients with diffuse large B‐cell lymphoma
Source: Cancer Med. 2023 Dec 22;13(1):e6856. doi: 10.1002/cam4.6856 (PMC10807600; doi:10.1002/cam4.6856)
Supplement: Supplementary file 4 — Table S4. [file CAM4-13-e6856-s004.docx]

**Table S4. Univariate and multivariate Cox analyses of OS in older patients with DLBCL.**

| Parameters | Univariate | | |  | Multivariate | | |
| --- | --- | --- | --- | --- | --- | --- | --- |
|  | *p* | HR | 95%CI |  | *p* | HR | 95%CI |
| Sex | 0.178 | 0.756 | 0.503-1.136 |  |  |  |  |
| Extranodal Sites | 0.029 | 1.633 | 1.052-2.535 |  | 0.121 | - | - |
| ECOG-PS | < 0.001 | 2.100 | 1.393-3.166 |  | 0.043 | 1.609 | 1.016-2.548 |
| Stage | 0.062 | 1.558 | 0.979-2.481 |  |  |  |  |
| B symptom | 0.461 | 1.170 | 0.770-1.778 |  |  |  |  |
| IPI | 0.057 | 1.600 | 0.987-2.593 |  |  |  |  |
| Hemoglobin | 0.038 | 1.549 | 1.025-2.343 |  | 0.330 | - | - |
| LDH | 0.691 | 1.087 | 0.719-1.643 |  |  |  |  |
| Albumin | < 0.001 | 2.208 | 1.470-3.315 |  | 0.006 | 1.888 | 1.198-2.975 |

Abbreviation: DLBCL, diffuse large B cell lymphoma; ECOG-PS, Eastern Cooperative Oncology Group performance status; IPI, International Prognostic Index; LDH, lactic dehydrogenase.
